# Online Mapping from Weight Matching Odometry and Highly Dynamic Point Cloud Filtering via Pseudo-Occupancy Grid

**DOI:** 10.3390/s25226872

**Published:** 2025-11-10

**Authors:** Xin Zhao, Xingyu Cao, Meng Ding, Da Jiang, Chao Wei

**Affiliations:** 1 China North Vehicle Research Institute, Norinco Group, Beijing 100072, China; 2 National Key Laboratory of Vehicular Transmission, Beijing Institute of Technology, Beijing 100081, China

**Keywords:** odometry, weight matching, object-level highly dynamic point cloud, pseudo-occupancy grid

## Abstract

Efficient locomotion in autonomous driving and robotics requires clearer visualization and more precise map. This paper presents a high accuracy online mapping including weight matching LiDAR-IMU-GNSS odometry and an object-level highly dynamic point cloud filtering method based on a pseudo-occupancy grid. The odometry integrates IMU pre-integration, ground point segmentation through progressive morphological filtering (PMF), motion compensation, and weight feature point matching. Weight feature point matching enhances alignment accuracy by combining geometric and reflectance intensity similarities. By computing the pseudo-occupancy ratio between the current frame and prior local submaps, the grid probability values are updated to identify the distribution of dynamic grids. Object-level point cloud cluster segmentation is obtained using the curved voxel clustering method, eventually leading to filtering out the object-level highly dynamic point clouds during the online mapping process. Compared to the LIO-SAM and FAST-LIO2 frameworks, the proposed odometry demonstrates superior accuracy in the KITTI, UrbanLoco, and Newer College (NCD) datasets. Meantime, the proposed highly dynamic point cloud filtering algorithm exhibits better detection precision than the performance of Removert and ERASOR. Furthermore, the high-accuracy online mapping is built from a real-time dataset with the comprehensive filtering of driving vehicles, cyclists, and pedestrians. This research contributes to the field of high-accuracy online mapping, especially in filtering highly dynamic objects in an advanced way.

## 1. Introduction

LiDAR mapping technology has made remarkable progress and is widely applied in various fields, including autonomous driving [1,2,3], SLAM systems [4,5,6] and robotic navigation [7,8,9,10]. Particularly in robotics [9,10,11], the synergistic integration of multi-sensors and computer vision has revolutionized the environmental perception capabilities of intelligent agents.

Recent research demonstrates that information fusion from a Global Navigation Satellite System (GNSS), an Inertial Measurement Unit (IMU), and a LiDAR sensor achieves high-precision mapping and localization in complex environments [12,13]. However, in reality, the presence of dynamic objects poses significant challenges to the accuracy of LiDAR mapping. Conventional point cloud matching algorithms typically assume a static environment. This assumption holds in most cases due to the short single-frame scanning time, and thus the impact of dynamic objects on odometry accuracy can be negligible [14]. Nevertheless, when objects move at a high speed, the resulting point cloud distortion severely disrupts matching precision, leading to accumulated localization errors. In some cases, moving objects introduced as “ghost artifacts” [15,16] appear into the generated map. The resulting map redundancy can severely mislead localization and path planning, decreasing accuracy and safety in navigation decision-making. Therefore, effectively suppressing and eliminating the side effects of highly dynamic point clouds on maps is of great significant in enhancing the reliability and practicality of LiDAR mapping systems.

According to the real-time performance of processing sensor data in dynamic environments, dynamic point cloud filtering algorithms can generally be categorized into two classes: online filtering and post-processing-based filtering. Online filtering methods typically select several consecutive point cloud frames as a reference and then compare them with the target frame in order to achieve superior real-time performance. For instance, RF-LIO [17], an extension of LIO-SAM [18], leverages the information from the initial pose of frontend odometry and local submaps to filter dynamic points on the current frame adaptively and iteratively, ultimately generating a static global map. ETH-Zurich’s ASL Lab proposed an end-to-end dynamic object detection framework [19], which is based on occupancy grids. This framework automatically labels dynamic objects and then trains them using 3D-MiniNet, enabling online detection and filtering of dynamic point clouds. Post-processing methods utilize all frames during the entire SLAM cycle as a reference, thus achieving higher accuracy and precision. The Peopleremover algorithm [20] constructs a regular voxel occupancy grid and then determines free voxels by traversing the lines of sight from the sensor to the measured points through the voxel grid. ERASOR [21], proposed by Hyungtae Lim et al., detects dynamic points by comparing region-wise occupancy ratios between the current scan and local submaps. The ratios exceeding a threshold are marked as dynamic. Removert [22], presented by Giseop Kim et al., built a novel 2D depth map by projecting a 3D point cloud with its neighboring submaps. By comparing the pixel depths at the same positions on the two maps, the shallower one is considered a dynamic point. This method outperforms manual annotations on the SemanticKITTI dataset [23], demonstrating an excellent filtering efficacy.

In real-world scenarios, to ensure accurate decision-making at high speeds requires prioritizing the real-time performance of maps. Simultaneously, at the same time, to optimize the effectiveness of decision-making and the robustness of motion behaviors, the map must be built with sufficiently high accuracy. In order to address it, therefore, this paper improves the precision of LiDAR-IMU-GNSS multi-sensor mapping by analyzing the geometric and reflective similarities of point cloud matching points to determine weight coefficients. It then employs a pseudo-occupancy grid-based method to filter object-level highly dynamic point clouds in real time, ultimately effectively enhancing accuracy and efficiency in mapping. Simulation experiments demonstrate that the accuracy of this odometry system surpasses that of LIO-SAM and FAST-LIO2 based on KITTI, UrbanLoco [24], and Newer College [25] (NCD) datasets. Furthermore, compared to the Removert and ERASOR algorithms, the proposed filtering method more effectively removes highly dynamic point clouds. The proposed algorithm is applied to a real-time dataset to build the map online with successful filtering of driving vehicles, cyclists, and pedestrians.

## 2. Algorithm Framework

**Figure 1:**
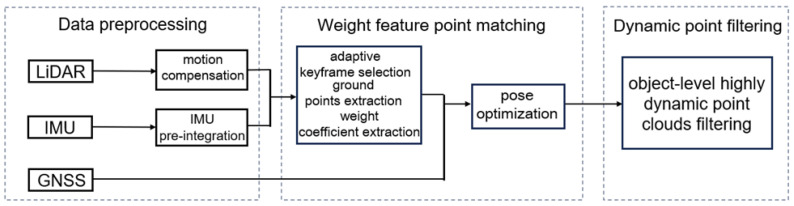
Algorithm framework.

This paper proposes an online mapping algorithm for the high accuracy multi-sensor fusion of LiDAR-IMU-GNSS and object-level highly dynamic point filtering. The framework comprises data preprocessing, weight feature point matching, and dynamic point filtering, as illustrated in Figure 1.

(i) Data preprocessing: Raw point cloud data from LiDAR, IMU, and GNSS are processed to generate synchronized and fused multi-sensor measurements. This includes IMU pre-integration, ground point segmentation using the Progressive Morphological Filter (PMF), and motion compensation of LiDAR point clouds.
(ii) Weight feature point matching: the weight coefficient of the Mahalanobis distance is determined by geometric and reflectance intensity similarities to obtain the optimized pose.
(iii) Dynamic point filtering: Dynamic objects are removed from the point cloud, and the map is built in real time using a pseudo-occupancy grid filtering algorithm.

## 3. Data Fusion for Weight Matching LiDAR-IMU-GNSS Odometry

### 3.1. IMU Pre-Integration

IMU pre-integration constructs constraints between LiDAR keyframes to suppress odometry drift and establish a pose graph that includes attitude, velocity, and position information. After acquiring IMU data and accounting for bias and noise, the predicted pre-integration values for attitude $R_{i}$, velocity $v_{i}$ and position $p_{i}$ between frames i and j are derived using a discrete-time integration method. Furthermore, their relative differences $\Delta R_{i,j}$, $\Delta v_{i,j}$, and $\Delta p_{i,j}$ are expressed in Equation (1),

(1) $$\left\{\begin{array}{c} \Delta R_{i,j}=R_{i}^{T}R_{j}=\prod_{k=i}^{j-1}Exp\left(\left({\tilde{\omega }}_{k}-b_{k}^{\omega }-n_{k}^{\omega }\right)\Delta t_{k}\right)\\ \Delta v_{i,j}=v_{j}-v_{i}=\sum_{k=i}^{j-1}\left(\left(R_{k}\left({\tilde{a}}_{k}-b_{k}^{a}-n_{k}^{a}\right)-g\right)\Delta t_{k}\right)\\ \Delta p_{i,j}=p_{j}-p_{i}=\sum_{k=i}^{j-1}\left(v_{k}\Delta t_{k}+\frac{1}{2}\left(R_{k}\left({\tilde{a}}_{k}-b_{k}^{a}-n_{k}^{a}\right)-g\right)\Delta t_{k}^{2}\right) \end{array}\right..$$

where ${\tilde{\omega }}_{k}$ and ${\tilde{a}}_{k}$ represent the angular velocity measured by the gyroscope and the linear acceleration by the accelerometer at the *k*th frame, respectively. $n_{k}^{\omega }$ and $n_{k}^{a}$ denote the bias noise of the angular velocity and acceleration at the *k*th frame. $b_{k}^{\omega }$ and $b_{k}^{a}$ are the measurement biases for the angular velocity and acceleration at the *k*th frame. $\Delta t_{k}$ is the time interval between the *k*th frame and the (*k* + 1)^th^ frame in the IMU data. g represents gravity. $Exp\left(.\right)$ denotes the mapping from a rotation vector to a rotation matrix. Therefore, the state at the (*k* + 1)^th^ frame can be predicted by integrating the state at the *k*th frame.

Assuming that the IMU biases $b_{k}^{\omega }=const$ and $b_{k}^{a}=const$ over a short period, the noise terms can be further simplified. Using the linear approximation from Lie group theory, we establish the relationship between the predicted $\Delta R_{i,j}$, $\Delta v_{i,j}$, and $\Delta p_{i,j}$ and the measured $\Delta {\tilde{R}}_{i,j}$, $\Delta {\tilde{v}}_{i,j}$, and $\Delta {\tilde{p}}_{i,j}$. The measured values are written as follows:

(2) $$\left\{\begin{array}{c} \Delta R_{i,j}=\Delta {\tilde{R}}_{i,j}Exp\left(-\delta \phi_{i,j}\right)\\ \Delta v_{i,j}=\Delta {\tilde{v}}_{i,j}-\delta v_{i,j}\\ \Delta p_{i,j}=\Delta {\tilde{p}}_{i,j}-\delta p_{i,j} \end{array}\right..$$

On the left-hand side of Equation (2) are the predicted values derived from the state variables, and on the right-hand side are the measured values obtained through IMU integration and a random noise term. To explicitly represent the measured values in Equation (2), it is necessary to derive the state transition equations for the random noise terms.

Equation (3) reveals that $\Delta R_{i,j}$ differs from $\Delta {\tilde{R}}_{i,j}$ by a noise term $Exp\left(-\delta \phi_{i,j}\right)$. By applying the operator $Log\left(.\right)$, which denotes the mapping from a rotation matrix to a rotation vector, the noise term can be linearly approximated. Retaining only the first-order term, the $\delta \phi_{i,j}$ is expressed as

(3) $$\delta \phi_{i,j}=-Log\left(\overset{\underset{j-1}{k=i}}{\prod }Exp\left(-\Delta {\tilde{R}}_{k+1,j}^{T}J_{r,k}n_{k}^{\omega }\Delta t_{k}\right)\right)\approx \sum_{k=i}^{j-1}\Delta {\tilde{R}}_{k+1,j}^{T}J_{r,k}n_{k}^{\omega }\Delta t_{k}=\Delta {\tilde{R}}_{k+1,j}^{T}\delta \phi_{i,j-1}+J_{r,j-1}n_{j-1}^{\omega }\Delta t_{j-1},$$

where $J_{r,k}$ is the Jacobian matrix between the *r*th frame and the *k*th frame, and similarly explanation to $J_{r,j-1}$. $n_{j-1}^{\omega }$ represents the (*j* − 1)^th^ bias noise of the angular velocity. $\Delta {\tilde{R}}_{k+1,j}^{T}$ represents the measured attitude difference between the (*k* + 1)^th^ frames and the j^th^ frame, and T is transpose. $\Delta t_{j-1}$ is the time interval between the (*j −* 1)^th^ frame and the *j*th frame in the IMU data.

Assuming that the noise terms of $\delta \phi_{i,j}$, $\delta v_{i,j}$, and $\delta p_{i,j}$ as well as the bias noise of the IMU, all follow zero-mean Gaussian distributions, we derive the following expression:

(4) $$\eta_{i,j}=\left[\begin{array}{c} \delta \phi_{i,j}\\ \delta v_{i,j}\\ \delta p_{i,j} \end{array}\right]∼N\left(0_{9\times 1},\Sigma_{\eta_{i,j}}\right)\;and\;\eta_{j}^{n}=\left[\begin{array}{c} n_{j}^{\omega }\\ n_{j}^{a} \end{array}\right]∼N\left(0_{6\times 1},\Sigma_{\eta_{j}^{n}}\right).\;$$

Similar procedures are applied in $\delta v_{i,j}$ and $\delta p_{i,j}$ terms. The state transition equation of $\eta_{i,j}$ is given by

(5) $$\eta_{i,j}=A_{j}\eta_{i,j-1}+B_{j}\eta_{j-1}^{n}A_{j}=\left[\begin{array}{ccc} \Delta {\tilde{R}}_{j-1,j}^{T} & 0 & 0\\ -\Delta {\tilde{R}}_{i,j-1}{\left({\tilde{a}}_{j-1}-b_{i}^{a}\right)}^{\wedge }\Delta t_{j-1} & I & 0\\ -\frac{1}{2}\Delta {\tilde{R}}_{i,j-1}{\left({\tilde{a}}_{j-1}-b_{i}^{a}\right)}^{\wedge }\Delta t_{j-1}^{2} & \Delta t_{j-1} & I \end{array}\right]\;and\;B_{j}=\left[\begin{array}{cc} J_{r,j-1}\Delta t_{j-1} & 0\\ 0 & \Delta {\tilde{R}}_{i,j-1}\Delta t_{j-1}\\ 0 & \frac{1}{2}\Delta {\tilde{R}}_{i,j-1}\Delta t_{j-1}^{2} \end{array}\right],\;\;$$

where I denotes the identity matrix. ∧ is the symbol for antisymmetric matrix. Consequently, the state transition equation for the covariance matrix of the noise term is derived as

(6) $$\Sigma_{\eta_{i,j}}=A_{j}\Sigma_{\eta_{i,j-1}}A_{j}^{T}+B_{j}\Sigma_{\eta_{j}^{n}}B_{k}^{T}.$$

The variation of IMU biases over time significantly affects the accuracy of measurements. By assuming that the pre-integrated measurements vary linearly with the IMU biases, we retain the first-order partial derivatives of the pre-integrated measurements with respect to the biases, so as to compensate for bias-induced errors in pre-integration.

(7) $$\left\{\begin{array}{c} \Delta {\tilde{R}}_{i,j}\left(b_{i}^{\omega }+\delta b_{i}^{\omega }\right)=\Delta {\tilde{R}}_{i,j}\left(b_{i}^{\omega }\right)Exp\left(\frac{\partial \Delta {\tilde{R}}_{i,j}}{\partial b_{i}^{\omega }}\partial b_{i}^{\omega }\right)\\ \Delta {\tilde{v}}_{i,j}\left(b_{i}^{\omega }+\delta b_{i}^{\omega },b_{i}^{a}+\delta b_{i}^{a}\right)=\Delta {\tilde{v}}_{i,j}\left(b_{i}^{\omega },b_{i}^{a}\right)+\frac{\partial \Delta {\tilde{v}}_{i,j}}{\partial b_{i}^{\omega }}\delta b_{i}^{\omega }+\frac{\partial \Delta {\tilde{v}}_{i,j}}{\partial b_{i}^{a}}\delta b_{i}^{a}\\ \Delta {\tilde{p}}_{i,j}\left(b_{i}^{\omega }+\delta b_{i}^{\omega },b_{i}^{a}+\delta b_{i}^{a}\right)=\Delta {\tilde{p}}_{i,j}\left(b_{i}^{\omega },b_{i}^{a}\right)+\frac{\partial \Delta {\tilde{p}}_{i,j}}{\partial b_{i}^{\omega }}\delta b_{i}^{\omega }+\frac{\partial {\tilde{p}}_{i,j}}{\partial b_{i}^{a}}\delta b_{i}^{a} \end{array}\right..$$

### 3.2. Ground Point Segmentation

The point cloud of the keyframe is extracted to reduce storage. In order to achieve the adaptive adjustment of the keyframe threshold, the parameter $d_{k}$ is determined based on the surrounding environment. Therefore, the adaptive keyframe threshold $di_{k}$ is expressed in Equation (8):

(8) $$di_{k}=\left\{\begin{array}{c} 5.0,\;\;\;d_{k}>20\\ 3.0,\;\;\;8<d_{k}\leq 20\\ 1.0,{\;\;\;\;d}_{k}\leq 8 \end{array}\right.,$$

where $d_{k}=\alpha d_{k-1}+\beta \bar{r}$, $\bar{r}$ is the median of the Euclidean distances from the point cloud of the current frame to the origin of the LiDAR. $d_{k-1}$ represents the openness of the scanning environment in the previous frame. α and β are constants, and α=0.9 and β=0.1 are chosen empirically in the experimental section.

During online mapping, the Progressive Morphological Filtering (PMF) algorithm is employed to segment ground points and non-ground points in each keyframe point cloud in real time.

PMF optimizes the window dimensions by gradually increasing the size of the filtering window. The update principles for the filtering window size $w_{k}$ and the height difference threshold $dh_{k}$ are presented in Equation (9).

(9) $$w_{k}=2b^{k}+1,dh_{k}=\left\{\begin{array}{c} dh_{0},w_{k}\leq 3\\ s\left(w_{k}-w_{k-1}\right)c+dh_{0},w_{k}>3\\ dh_{max},dh_{k}>dh_{max} \end{array}\right.,$$

where $w_{k}$ denotes the window size at the *k*th iteration. b represents the initial window size. $dh_{0}$ and $dh_{max}$ refer to the initial and maximum height difference thresholds, respectively. s indicates the slope, and $s=2d{h\left(w_{k}-w_{k-1}\right)}_{max}$. c stands for the grid size.

### 3.3. Motion Compensation of LiDAR Point Clouds

At high vehicle speeds, motion distortion compensation for point clouds becomes essential. When IMU data is available, IMU-based motion compensation in continuous time is used. Otherwise, in the case of IMU data dropout, the motion compensation method based on a constant velocity model is used. Assuming that the current frame maintains the same motion state as the previous LiDAR frame, the LiDAR pose at each timestamp within the current frame can be calculated through interpolation, as expressed below,

(10) $$\left\{\begin{array}{c} R^{*}=R_{i-1}\cdot Exp\left(\mu \Delta \phi \right)\\ \Delta \phi =Log\left(R_{i-2}^{T}\cdot R_{i-1}^{T}\right)\\ p^{*}=p_{i-1}+\mu \left(p_{i-1}-p_{i-2}\right)\\ \mu =\frac{t^{*}-t_{i-1}}{t_{i-1}-t_{i-2}} \end{array}\right.,$$

where $R^{*}$ and $p^{*}$ denote the query point’s attitude and position. $t^{*}$ represents the current timestamp.

### 3.4. Weight Feature Point Matching Method Based on Geometric-Reflectance Intensity Similarity

The Mahalanobis distance between points is commonly used as the criterion for feature point matching and for obtaining the optimized pose. However, assigning the same weight to each matched point fails to accurately reflect the varying influence of uncertainty across different matched point pairs. During the point matching process, the weight of false matches should be significantly reduced. In addition, the positional uncertainty of the matched points introduces noise, and thus the weight of such points should decrease as their uncertainty increases.

To enhance both point matching accuracy and calculational efficiency, an eigenvalue-based approach is employed to extract planar feature points. Accordingly, the centroid and the covariance matrix of the point distribution around the point i is given in Equation (11) based on the KD-Tree method.

(11) $$\left\{\begin{array}{c} p_{center}=\frac{1}{N}\sum_{j=1}^{N}p_{j}\\ C_{i}=\frac{1}{N-1}\sum_{i=1}^{N}\left(\left(p_{i}-p_{center}\right)\cdot {\left(p_{i}-p_{center}\right)}^{T}\right) \end{array}and\left\{\begin{array}{c} C_{i}=U_{i}\cdot \sum_{i}\cdot V_{i}^{T}\\ \sum_{i}=\left[\begin{array}{ccc} \lambda_{0} & 0 & 0\\ 0 & \lambda_{1} & 0\\ 0 & 0 & \lambda_{2} \end{array}\right]\\ \lambda_{0}\geq \lambda_{1}\geq \lambda_{2}\geq 0 \end{array}\right.,\right.$$

where $\lambda_{0}$, $\lambda_{1}$, and $\lambda_{2}$ are the three eigenvalues of the covariance matrix.

If eigenvalues satisfy Equation (12), the point i is a planar feature point.

(12) $$\left\{\begin{array}{c} \frac{\lambda_{2}}{\lambda_{0}+\lambda_{1}}<\rho_{0}\\ \frac{\lambda_{0}-\lambda_{1}}{\lambda_{0}+\lambda_{1}}<\rho_{1} \end{array}\right.,$$

where $\rho_{0}$ and $\rho_{1}$ are thresholds.

In order to reduce incorrect matching, the similarity between two feature points should be taken into account. By assigning different weights to each point, the residual function for point cloud matching can be formulated. In this section, a weight feature point matching method based on the geometric-reflectance intensity similarity of point clouds is proposed.

The geometric similarity is defined by the positional relationship between the normal vectors n of the planar feature point i in one point cloud and j in the target point cloud, separately. Note that $\lambda_{2}$ is the smallest eigenvalue; its eigenvector $v_{2}$ is thus approximately equivalent to the normal vector of the corresponding plane. The subscript i and j correspond to the points i and j. Therefore, the geometric similarity $S_{G,i}$ can be expressed as

(13) $$S_{G,i}=\frac{n_{i}^{T}n_{j}}{\left\|n_{i}\right\|\left\|n_{j}\right\|}\approx \frac{v_{2,i}^{T}v_{2,j}}{\left\|v_{2,i}\right\|\left\|v_{2,j}\right\|}.$$

The reflection intensity of matched points exhibits similar distributions and is thus chosen as the second similarity. We assume that the reflection intensity around points i and j follows normal distributions $N_{i}\left(\mu_{i},\sigma_{i}^{2}\right)$ and $N_{j}\left(\mu_{j},\sigma_{j}^{2}\right)$, in which $\mu_{i}$, $\sigma_{i}$ and $\mu_{j}$, $\sigma_{j}$ represent the mean value and standard deviation for points i and j, respectively. The distances from the two points to the sensor center are $r_{i}$ and $r_{j}$, with $r_{j}=\kappa r_{i}$ (where κ is the scale factor), with their reflection intensity satisfies scaling law as well. Therefore, by applying Kullback–Leibler (KL) divergence theory to the reflection intensity of matched points [26], a modified KL divergence model is yielded based on Lambertian theory:

(14) $$\left\{\begin{array}{c} \mu_{j}=\kappa^{2}\mu_{i}\\ \sigma_{j}^{2}=\kappa^{4}\sigma_{i}^{2}\\ KL_{sym}=\frac{\mu_{i}^{2}}{4\sigma_{i}^{2}}\left(\frac{1}{\kappa^{4}}+1\right){\left(1-\kappa^{2}\right)}^{2}+\frac{1}{4\kappa^{4}}+\frac{\kappa^{4}}{4}-\frac{1}{2} \end{array}\right..$$

Since the weight coefficient is not larger than 1, a Gaussian function is used to normalize the Kullback–Leibler (KL) divergence, thereby obtaining the reflection intensity similarity $S_{I}$ in Equation (15).

(15) $$S_{I}=\exp\left(-\frac{{\left(KL_{sym}^{norm}\right)}^{2}}{2\eta^{2}}\right),$$

where η is the Gaussian scale factor.

To avoid the sparsity error, the average planarity $S_{p}$ of the points i and j is introduced as the third weight coefficient.

(16) $$S_{p}=\frac{1}{2}\left(\frac{\lambda_{1}^{i}-\lambda_{2}^{i}}{\lambda_{0}^{i}}+\frac{\lambda_{1}^{j}-\lambda_{2}^{j}}{\lambda_{0}^{j}}\right).$$

Eventually, the weight w of the points i and j is defined as

(17) $$w=S_{G}\cdot S_{I}\cdot S_{p}.$$

To ensure the real-time performance of the odometer, the planar feature point cloud of the current frame is matched with that of the local submap in the keyframe. The transformation matrix is obtained by minimizing the weighted Mahalanobis distance derived from the local geometric characteristics, as shown in Equation (18).

(18) $$\left\{\begin{array}{c} R,t=\underset{R,t}{argmin}\sum_{i=0}^{N}w_{i}r_{L^{i}}\\ r_{L^{i}}=d_{i}^{T}{\left({\tilde{C}}_{i}^{M}+T{\tilde{C}}_{i}^{P}T^{T}\right)}^{-1}d_{i}\\ T=\left[\begin{array}{cc} R & t\\ 0_{1\times 3} & 1 \end{array}\right],{\tilde{C}}_{i}=\left[\begin{array}{cc} C_{i} & 0_{3\times 1}\\ 0_{1\times 3} & 1 \end{array}\right] \end{array}\right.,$$

where $r_{L^{i}}$ represents the Mahalanobis distance calculated by the GICP algorithm. $d_{i}$ represents the transformation error term between the current frame and the local submap of the keyframe. ${\tilde{C}}_{i}$ represents the covariance matrix of the points. The superscripts P and M represent the covariance matrices of the current frame and the submap, respectively. For the covariance matrix $C_{i}=V\Lambda V^{T}$, since all the feature points lie on a plane, the diagonal elements of Λ are replaced with (1,1,*ϵ*), where *ϵ* is a very small constant, to regularize the covariance matrix Λ.

## 4. Online Filtering Method for Highly Dynamic Point Clouds

If the set of keyframe point clouds is denoted as F, after extracting the ground points, the points are divided into ground points G and non-ground points P. Furthermore, the point cloud set obtained by transforming each keyframe point cloud into the global coordinate system and then splicing them together is the map M. If the highly dynamic point cloud map is represented as $M_{d}$, then the static point cloud map can be expressed as $M_{s}=M-M_{d}$. Removing point clouds from a map directly may result in point cloud loss and over-filtering, but removing only the point clouds from one or several frames allows the point clouds from other frames to compensate for the loss in the map. Therefore, we obtain the static map by filtering the object-level highly dynamic point clouds at each keyframe, as shown in Equation (19).

(19) $$M_{s}={⋃}_{k=1}^{N}\left(G_{k}+P_{k}-{⋃}_{I_{k}^{d}\in I_{k}}I_{k}^{d}\right),$$

where $G_{k}$ is the ground point cloud of the *k*th keyframe, which is regarded as the static point cloud. $I_{k}^{d}$ represents the object-level highly dynamic point cloud set of the *k*th keyframe. $P_{k}$ denotes all object-level point clouds in the *k*th keyframe.

Objects that generate highly dynamic points, such as pedestrians, vehicles, and so forth, temporarily occupy a certain area in the map for a short time, while static points remain persistently fixed in place. Therefore, by comparing the grid occupancy between the current frame’s point cloud and the map, we can identify regions occupied by dynamic objects. Consequently, non-ground points within these dynamic regions can be classified as highly dynamic points.

First, to eliminate the influence of outliers in the point cloud, a pass-through filter is applied to extract the region of interest. The filtering range is set from $h_{min}$ to $h_{max}$. Then, the point cloud of the region of interest in the current keyframe is represented as

(20) $$P_{k}^{int}=\left\{\left.p\in P_{k}\right|h_{max}{\geq h}_{p}\geq h_{min}\right\}.$$

The grid obtained from the point cloud of the region of interest $P_{k}^{int}$ in the global coordinate system is $G_{k}$. Thus the local submap can be represented by Equation (21).

(21) $$M_{k}^{sub}=\left\{\left.p\in M\right|\Gamma \left(p\right)\in G_{k}\right\},$$

where the grid projection function $\Gamma \left(p\right)$ is defined as $\Gamma \left(p\right)=\left(x,y\right)=(floor\left(x\cdot res_{x}\right),floor\left(y\cdot res_{y}\right))$, $res_{x}$ and $res_{y}$ are the grid resolutions in the X and Y dimensions, respectively. $\left(x,y\right)$ is the index of the grid corresponding to the point p.

The pseudo-occupancy rate is defined as the difference between the maximum and minimum heights of the point cloud within the grid. Therefore, the rates $\Delta h_{i,j}^{k}$ and $\Delta h_{i,j}^{M_{k}^{sub}}$ of the current keyframe k and the local submap are expressed as follows:

(22) $$\begin{array}{c} \Delta h_{i,j}^{k}=\max\left(z_{G_{i,j}}\right)-z_{k,i,j}^{low}\\ \Delta h_{i,j}^{M_{k}^{sub}}=\max\left(z_{G_{i,j}}\right)-z_{i,j}^{M_{k}^{sub},low}, \end{array}$$

where $G_{i,j}$ represents the grid with index $\left(i,j\right)$; $z_{i,j}^{M_{k}^{sub},low}$ and $z_{k,i,j}^{low}$ represent the minimum values of the point cloud heights of the grid with index $\left(i,j\right)$ in the local submap and the current keyframe k, respectively.

The pseudo-occupancy ratio determines the occupancy status of dynamic point clouds in the current frame’s grid, and it is defined as the ratio between the pseudo-occupancy rate of the current frame and the submap grid, as shown in Equation (23).

(23) $$\frac{\Delta h_{i,j}^{k}}{\Delta h_{i,j}^{M_{k}^{sub}}}>\tau_{h},$$

where $\tau_{h}$ is the threshold for the pseudo-occupancy ratio. If the pseudo-occupancy ratio exceeds $\tau_{h}$, the grid $\left(i,j\right)$ in the current frame is considered to contain dynamic point clouds.

The joint distribution of the binary states m for pseudo-occupancy grids is expressed as follows:

(24) $$p\left(\left.m\right\rfloor z_{1:t}\right)=\prod_{i}p\left(\left.m_{i}\right\rfloor z_{1:t}\right),$$

where $m_{i}$ represents the occupancy state of the *i*th grid, and $z_{1:t}$ denotes all observations up to frame t, where each observation corresponds to the difference between the scan at frame i and the map.

We update the probability information in the map grid according to Bayes’ rule; that is,

(25) $$\log it\left(p\left(\left.m_{i}\right|z_{1:t}\right)\right)=\log it\left(p\left(\left.m_{i}\right|z_{1:t-1}\right)\right)+\log it\left(p\left(\left.m_{i}\right|z_{t}\right)\right)-\log it\left(p\left(m_{i}\right)\right),$$

where $\log it\left(p\left(x\right)\right)$ defines the occupancy and non-occupancy of the grid, and $\log it\left(p\left(x\right)\right)=\log\left(p\left(x\right)/\left(1-p\left(x\right)\right)\right)$.

The update rule for $\log it\left(p\left(x\right)\right)$ is as follows:

(26) $$\log it\left(p\left(\left.m_{i,j}\right|z_{k}\right)\right)=\left\{\begin{array}{c} \kappa \log it\left(p\left(\left.m_{i,j}\right|z_{k}\right)\right),\frac{\Delta h_{i,j}^{k}}{\Delta h_{i,j}^{M_{k}^{sub}}}>\tau_{h}\\ \log it\left(p\left(\left.m_{i,j}\right|z_{k}\right)\right),\frac{\Delta h_{i,j}^{k}}{\Delta h_{i,j}^{M_{k}^{sub}}}<\tau_{h},\frac{G_{i,j}^{k}}{{P_{k,i,j}^{int}}_{gd}}>\tau_{gd}\\ \log it\left(p_{0}\right) \end{array}\right.,$$

In Equation (26), for $\frac{\Delta h_{i,j}^{k}}{\Delta h_{i,j}^{M_{k}^{sub}}}>\tau_{h}$, $\log it\left(p\left(\left.m_{i,j}\right|z_{k}\right)\right)$ is increased by a gain factor κ (κ>1). Therefore, the updated probability value $\log it\left(p\left(\left.m_{i,j}\right|z_{k}\right)\right)$ increases, and $\log it\left(p\left(\left.m_{i,j}\right|z_{k}\right)\right)>\log it\left(p_{0}\right)$. For $\frac{\Delta h_{i,j}^{k}}{\Delta h_{i,j}^{M_{k}^{sub}}}<\tau_{h}$ and $\frac{G_{i,j}^{k}}{P_{k,i,j}^{int}}>\tau_{gd}$, the grid is still likely occupied by dynamic points and is updated using $\log it\left(p\left(\left.m_{i,j}\right|z_{k}\right)\right)$. Otherwise, $\log it\left(p\left(x\right)\right)$ retain its initial value $\log it\left(p_{0}\right)$. $p_{0}$ represents an initial prior probability.

In order to avoid deleting objects with ambiguous boundaries by mistake, a curved voxel point cloud clustering algorithm is employed [27]. This algorithm detects the point cloud corresponding to the object in the map, enabling subsequent object-level segmentation of the highly dynamic point cloud in the map. If all objects containing dynamic grids are filtered out without considering their dynamic attributes, static objects may be inadvertently removed. Therefore, an object dynamic score is defined for each object point cloud, as shown in Equation (27), serving as a dynamic evaluation criterion to accurately distinguish and separate the highly dynamic object point cloud completely.

(27) $$Score_{i}=\frac{1}{Num\left({Cl}_{i}\right)}\sum_{k=1}^{Num\left({Cl}_{i}\right)}\log it\left(f\left(G\left(p_{k}^{{Cl}_{i}}\right)\right)\right),$$

where $Num\left({Cl}_{i}\right)$ represents the number of point clouds of the *i*th object. $G\left(p_{k}^{{Cl}_{i}}\right)$ represents the grid corresponding to each point in the object ${Cl}_{i}$. The function $f\left(.\right)$ returns the corresponding value based on the probability value of the grid. The definition is as follows:

(28) $$f\left(G\left(p\right)\right)=\left\{\begin{array}{c} p\left(\left.m_{i}\right|z_{1:t}\right),p\left(\left.m_{i}\right|z_{1:t}\right)\geq p_{0}\\ p_{-} \end{array}\right.,$$

where the true probability of the grid is returned when the grid probability exceeds the initial value $p_{0}$. Otherwise, a constant $p_{-}$ ($p_{-}<1$) is returned. It should be noted that when the grid is always occupied by a static object, the probability of the grid remains $p_{0}$, and its log odds value of the grid remains $\log it\left(p_{0}\right)$. When both static and dynamic objects occupy the grid, the presence of $p_{-}$ reduced the object dynamic score, thereby decreasing the probability of mistakenly removing the static object. The final result is dynamic object point cloud.

(29) $$D=\left\{\left.{Cl}_{i}\right|\log it^{-1}\left(Score_{i}\right)>\tau_{score}\right\},$$

where $\log it^{-1}$ is the inverse of the log odds function, converting the log odds back to a probability value. $\tau_{score}$ is the adaptive probability threshold for dynamic objects. ${Cl}_{i}$ is the point cloud cluster.

When performing point cloud clustering, two objects located close to each other may be mistakenly identified as a single cluster. As a result, static objects within may be removed incorrectly. Since such point clouds generally occupy a large number of grids but contain a small proportion of highly dynamic grids, they can be distinguished based on the total number of occupied grids and the proportion of dynamic grids, as shown in Equation (30).

(30) $$\left\{\begin{array}{c} Num\left(G\left({Cl}_{i}\right)\right)>\tau_{grid}\\ 0<\frac{Num\left(G_{dynmic}\left({Cl}_{i}\right)\right)}{Num\left(G\left({Cl}_{i}\right)\right)}<\mu_{dynamic} \end{array}\right..$$

The weights of point cloud features are considered by LiDAR-IMU-GNSS odometry when calculating the Mahalanobis distance between the matching point clouds.

$Num\left(G\left({Cl}_{i}\right)\right)$ represents the number of grids occupied by the point cloud cluster ${Cl}_{i}$. $\tau_{grid}$ represents the minimum threshold of occupied grids. $Num\left(G_{dynmic}\left({Cl}_{i}\right)\right)$ represents the number of dynamic grids in the point cloud cluster ${Cl}_{i}$. $\mu_{dynamic}$ represents the maximum proportion threshold of dynamic grids.

After removing highly dynamic grids from the under-segmented point cloud, the static map is ultimately established.

## 5. Algorithm Validation

### 5.1. Accuracy of Proposed Odometry Systems

For the sake of accurately validating the proposed weight matching LiDAR-IMU-GNSS odometry, our method is tested on the KITTI, UrbanLoco [24], and Newer College [25] (NCD) datasets, and compared with the LIO-SAM and FAST-LIO2 methods.

**Figure 2:**
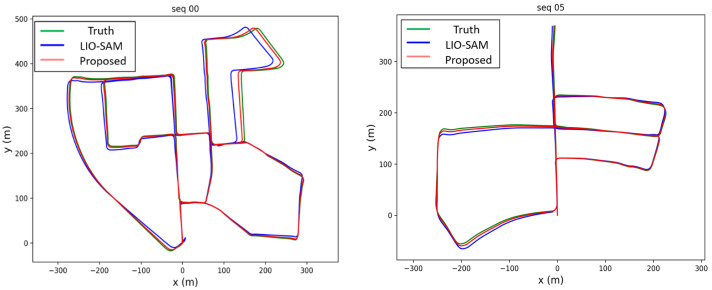
The 00 and 05 sequences of the KITTI dataset. The ground truth trajectory is shown in green, the LIO-SAM trajectory in blue, and the trajectory of the proposed algorithm in red.

We present the trajectory comparison between the proposed algorithm and the LIO-SAM algorithm on 00, 05, 09, and 10 sequences of the KITTI dataset. Figure 2 illustrates the typical results from 00 and 05 sequences. The ground truth trajectory is shown in green, the LIO-SAM trajectory in blue, and the trajectory of the proposed algorithm in red. Clearly, the trajectory produced by the proposed algorithm is significantly closer to the ground truth, indicating that it achieves higher accuracy than the LIO-SAM algorithm.

The root-mean-squared error (RMSE) of the absolute trajectory error (ATE) was used to evaluate the error across the entire trajectory. The RMSE is defined as follows:

(31) $$RMSE_{ATE}=\sqrt{\frac{1}{N}\sum_{i=1}^{N}{\left\|trans\left(T_{gt}^{-1}T_{est,i}\right)\right\|}^{2}},$$

where N represents the number of frames in the dataset. trans(.) denotes the extraction of the translational component of the pose, and the Euclidean norm is used to compute the vector magnitude in the RMSE calculation.

Table 1 summarizes the RMSE values for three algorithms across subsets of the KITTI, UrbanLoco, and NCD datasets. The smaller the error is, the better the result is. The results indicate that the proposed algorithm achieves higher accuracy than the other two open-source algorithms in most datasets, demonstrating superior performance.

### 5.2. Comparison of Highly Dynamic Point Cloud Filtering Algorithm

**Figure 3:**
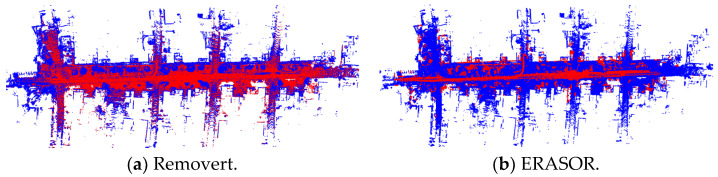

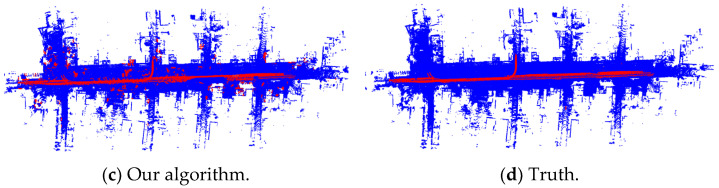
Three algorithms on sequence 00 of the SemanticKITTI dataset. The red points represent the highly dynamic object point clouds, and the blue ones represent the static point clouds.

**Figure 4:**
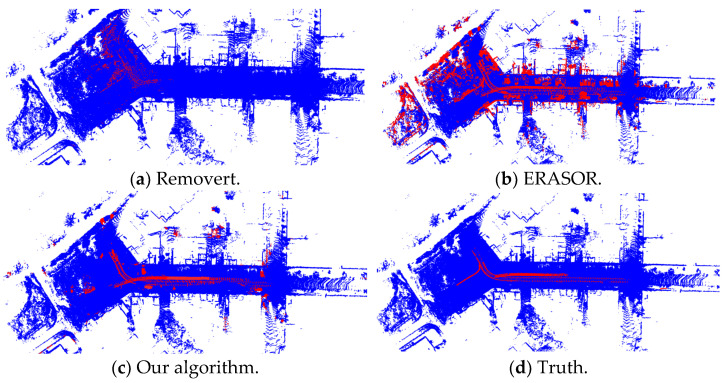
Three algorithms on sequence 05 of the SemanticKITTI dataset. The red points represent the highly dynamic object point clouds, and the blue ones represent the static point clouds.

Figure 3 and Figure 4 show the visualization results of the proposed highly dynamic point cloud filtering algorithm, the Removert and ERASOR algorithms based on sequence 00 and 05 of the SemanticKITTI dataset, meantime, the truth is given as well. In the figures, the red point clouds represent the highly dynamic object point clouds detected by each algorithm, while the blue ones represent the static point clouds.

In Figure 3a,b, compared with the ground truth, both the Removert and ERASOR algorithms effectively filter out dynamic points, but they incorrectly remove a large number of static points. This results in an excessive number of false-positive detections, ultimately producing a relatively sparse map. In contrast, the method proposed in this paper ensures high detection precision while generating fewer false positive points. In Figure 4, using the sequence 05 dataset, the Removert algorithm achieves a relatively low detection precision, and the ERASOR algorithm has a relatively high proportion of false positives, while the proposed algorithm outperforms both on this dataset.

Furthermore, using sequences 00, 01, 05, and 07 from the SemanticKITTI dataset, which contain numerous dynamic objects, the accuracy of the proposed highly dynamic point cloud filtering algorithm is compared with the Removert and ERASOR algorithms. Table 2 presents the Preservation Rate (PR), Recall Rate (RR), and F_1_ score of the proposed highly dynamic point cloud filtering module in comparison with the Removert and ERASOR algorithms. The three metrics are defined as follows:

(32) $$\left\{\begin{array}{c} PR=\frac{TP}{TP+FP}\\ RR=\frac{TP}{TP+FN}\\ F_{1}=2\times \frac{PR\times RR}{PR+RR} \end{array}\right.$$

where TP (True Positive) represents the number of samples that are correctly detected as dynamic object categories. FP (False Positive) represents the number of samples that are actually static but incorrectly detected as dynamic categories. FN (False Negative) represents the number of samples that are actually dynamic but incorrectly detected as static categories. The values of all three metrics range from 0 to 1, with values closer to 1 indicating better performance.

Table 2 presents the performance of Removert, ERASOR, and the proposed method across three metrics on the SemanticKITTI dataset. The results demonstrate that the proposed highly dynamic point cloud filtering algorithm achieves high accuracy, with fewer missed and false detections. It can effectively filter highly dynamic objects in single-frame scanned point clouds and eliminate the “long trailing shadows” in the map caused by moving objects.

### 5.3. Online Filtering Experiment

A dataset at an intersection on the campus of Beijing Institute of Technology (BIT) was obtained to evaluate the proposed online highly dynamic point cloud filtering algorithm in this paper. The intersection contains numerous highly dynamic objects, such as heavy traffic and a large pedestrian flow.

In the experiment, the Wuling Hongguang miniEV serves as the test vehicle, equipped with an environmental perception system including three sensors. The Robosense Helio-32 mechanical LiDAR offers a 360° horizontal field of view and a 70° vertical field of view, with the ranging accuracy within 2 cm and a scanning frequency of 10 Hz. The GW-NAV100B is a dual-antenna, high-precision MEMS integrated navigation system. It incorporates a built-in three-axis gyroscope, a three-axis accelerometer, and a four-mode (BD/GPS/GLONASS/GALILEO) receiver, enabling it to measure the velocity, position, and attitude of the test vehicle. Xsens Mti-630 is a 9-axis IMU with a 100 Hz output frequency. During the data collection process, the total duration of the experiment was approximately 1000 s, covering a distance of about 3000 m. To construct local submaps, the data was divided equally into five segments, with each being approximately 600 m long. The parameters of the Robosense Helio-32 mechanical LiDAR and GW-NAV100B are listed in Table 3.

**Figure 5:**
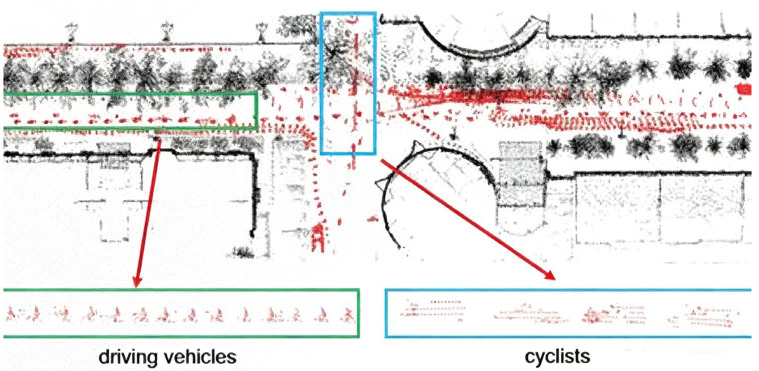
Scenario 1 and highly dynamic objects. The blue and green boxes enclose the detailed point cloud maps of vehicles and cyclists.

**Figure 6:**
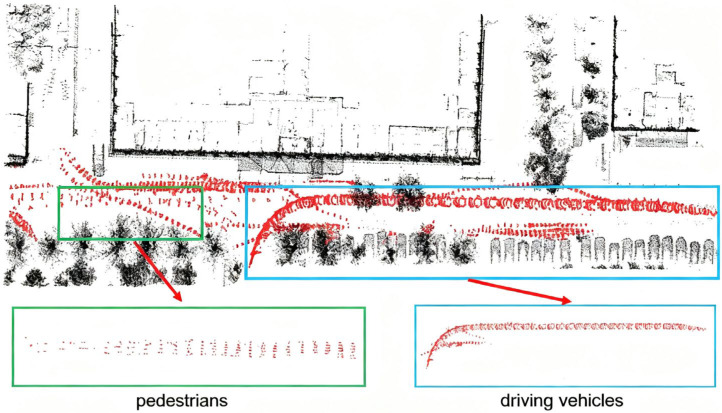
Scenario 2 and highly dynamic objects. The blue and green boxes enclose the detailed point cloud maps of vehicles and pedestrians.

Two typical highly dynamic scenarios are shown in Figure 5 and Figure 6. The black point clouds represent static environmental point clouds, while the red point clouds represent the highly dynamic objects detected by the method proposed in this paper.

In Figure 5 and Figure 6, it can be observed that at the intersection, there are a large number of point cloud “long trailing shadows” caused by pedestrians, cyclists, and vehicles, which degrades the quality of the point cloud map. Typical highly dynamic objects, such as driving vehicles, cyclists and pedestrians, are clearly identifiable. In Figure 5, the blue and green boxes, respectively, enclose the detailed point cloud maps of vehicles and cyclists; in Figure 6, the blue and green boxes, respectively, enclose the detailed point cloud maps of vehicles and pedestrians. As demonstrated in Figure 5 and Figure 6, the online highly dynamic point cloud filtering algorithm proposed in this paper can effectively detect major highly dynamic objects such as pedestrians, cyclists, and vehicles in highly dynamic scenarios.

## 6. Conclusions

This paper improves the mapping method with weight matching LiDAR-IMU-GNSS odometry and a proposed online highly dynamic point cloud filtering algorithm. The weight matching LiDAR-IMU-GNSS odometry includes IMU pre-integration, ground point extraction, motion compensation, and weight feature point matching. Specifically, within the weight matching LiDAR-IMU-GNSS odometer, a method for point cloud keyframe selection and submap construction based on an adaptive threshold is designed. Ground point clouds and planar point clouds are extracted separately using the PMF and KD-Tree methods. A weight feature point matching method based on geometric-reflectance intensity similarity is proposed to improve point cloud matching. Subsequently, by using the pseudo-occupancy ratio between the current frame point cloud and the previous local submap as an observation, grid probability values are updated to obtain the distribution of dynamic features in the map. In addition, the curved voxel point cloud clustering algorithm is used to obtain object-level point cloud clusters. This approach enables comprehensive filtering of highly dynamic object-level point clouds.

The higher accuracy of the weight matching LiDAR-IMU-GNSS odometer is valid in the KITTI, UrbanLoco, and NCD datasets in comparison with the LIO-SAM and FAST-LIO2 methods. The proposed highly dynamic point cloud filtering algorithm outperforms the Removert and ERASOR algorithms based on the sequences from the SemanticKITTI dataset. Finally, online mapping with highly dynamic point cloud filtering is applied in a real-time dataset, effectively detecting typical highly dynamic objects such as driving vehicles, cyclists, and pedestrians. We hope this research will contribute to advancements in high-accuracy online mapping with dynamic object filtering and promote the development of navigation and locomotion in autonomous driving and robotics.

## Figures and Tables

**Table 1:** The RMSE of three algorithm frameworks on datasets (RMSE/m).

Dataset	LIO-SAM	FAST-LIO2	Our Method
00	5.8	3.7	**1.1**
01	11.3	**10.8**	10.9
02	**11.8**	13.2	12.9
03	**-**	-	-
04	1.2	1.0	**0.9**
05	3.0	2.8	**2.5**
06	**1.0**	1.3	1.1
07	1.2	**1.1**	**1.1**
08	4.4	**3.9**	**3.9**
09	4.3	4.8	**2.1**
10 UrbanLoCo-CA-1 UrbanLoCo-CA-2 UrbanLoCo-HK-1 UrbanLoCo-HK-2 NCD-long-13	2.4 5.295 11.635 1.342 1.782 0.187	1.7 10.943 7.901 1.196 1.802 0.194	**1.5** **4.615** **7.189** **1.159** **1.768** **0.163**
NCD-long-14	0.195	0.212	**0.185**
NCD-long-15	**0.162**	0.173	0.169

**Table 2:** Results of SemanticKITTI datasets.

Dataset	Method	PR	RR	F_1_
00	Removert	86.8	90.6	0.88
	ERASOR	93.9	97.0	0.95
	Our Algorithm	98.7	98.5	0.98
01	Removert	95.8	57.0	0.71
	ERASOR	91.8	94.3	0.93
	Our Algorithm	96.8	94.6	0.95
05	Removert	86.9	87.8	0.87
	ERASOR	88.7	98.2	0.93
	Our Algorithm	97.5	96.3	0.96
07	Removert	80.6	98.8	0.88
	ERASOR	90.6	99.2	0.948
	Our Algorithm	96.6	98.9	0.977

**Table 3:** Parameters of Robosense Helio-32 mechanical LiDAR and GW-NAV100B.

Robosense Helio-32			GW-NAV100B	
Parameters		Values	Parameters	Values
vertical field	range	+15°~−55°	frequency	100 Hz
		2°(+15°~+7° and −8°~−55°)		(10 Hz for GNSS)
	resolution	1.5(+7°~+4°)	position resolution	0.01 m in horizonal field
		1.33(+4°~−8°)		0.15 m in vertical field
horizontal field	range	360°	velocity resolution	0.03 m/s
	resolution	0.2°		0.2° in roll
			attitude resolution	0.2° in pitch
				0.1° in yaw

## Data Availability

The data that support the findings of this study are available from the corresponding author upon reasonable request.
